# 非小细胞肺癌N2亚分期研究进展

**DOI:** 10.3779/j.issn.1009-3419.2022.101.53

**Published:** 2022-12-20

**Authors:** 永兴 包, 周光 惠

**Affiliations:** 1 100021 北京，国家癌症中心/国家肿瘤临床医学研究中心/中国医学科学院北京协和医学院肿瘤医院放疗科 Department of Radiation Oncology, National Cancer Center/National Clinical Research Center for Cancer/Cancer Hospital, Chinese Academy of Medical Sciences and Peking Union Medical College, Beijing 100021, China; 2 100021 北京，国家癌症中心/国家肿瘤临床医学研究中心/中国医学科学院北京协和医学院肿瘤医院特需医疗部 Department of VIP Medical Services, National Cancer Center/National Clinical Research Center for Cancer/Cancer Hospital, Chinese Academy of Medical Sciences and Peking Union Medical College, Beijing 100021, China

**Keywords:** 肺肿瘤, N2, 亚分期, 总生存期, 无病生存期, 复发模式, Lung neoplasms, N2, Sub-staging, Overall survival, Disease-free survival, Failure patterns

## Abstract

N2期非小细胞肺癌的异质性较大，国际肺癌研究学会根据预后差异将其划分为三个亚分期，即N1淋巴结阴性的单站N2淋巴结转移（N2a1）、N1淋巴结阳性的单站N2淋巴结转移（N2a2）以及多站N2淋巴结转移（N2b）。目前的证据显示，N2亚分期可以更好地区分非小细胞肺癌的总生存期（overall survival, OS）、无病生存期（disease-free survival, DFS）及复发模式。N2a1人群的OS及DFS更接近多站N1淋巴结转移（N1b）；N2a2人群的OS及DFS介于N2a1及N2b之间；N2b人群的OS及DFS较差，目前的证据尚不支持将N2b期进一步细分。尽管危险度不同，但N2a1、N2a2及N2b三个亚分期的主要复发模式均为远处转移，且远处转移的风险依次升高。N2a1人群的局部区域复发风险较低，术后放疗不能改善其局部区域复发；而N2a2和N2b人群的局部区域复发风险相似，术后放疗可以显著改善这两个人群的局部区域复发，使之降低至与N2a1人群相近的水平。

## Abstract (en)

Patients with N2 non-small cell lung cancer (NSCLC) were heterogeneous groups and required further stratification. The International Society for the Study of Lung Cancer (IASLC) divided N2 into three sub-stages: N2 at a single station without N1 involvement (N2a1), N2 at a single station with N1 involvement (N2a2), and N2 at multiple stations (N2b). These new descriptors significantly distinguished the overall survival (OS), disease-free survival (DFS), and recurrence pattern of patients with different N2 sub-stages. The OS and DFS of N2a1 were not sufficiently distinguished from N1 at multiple stations (N1b). The OS and DFS of N2a2 were intermediate between those of N2a1 and N2b. Current evidence did not support the further subdivision of the N2b. The main recurrence pattern of N2a1, N2a2 and N2b were distant metastasis, and the risks of distant metastasis increased successively. N2a1 patients were at low risk of locoregional recurrence, which could not be reduced by postoperative radiotherapy (PORT). N2a2 and N2b patients had a similar higher risk of locoregional recurrence, which could be reduced to a similar level of N2a1 patients by PORT.

肺癌的发病率及死亡率均位居恶性肿瘤前列，非小细胞肺癌（non-small cell lung cancer, NSCLC）约占肺癌的80%^[1]^。肿瘤原发灶-淋巴结-转移（tumor-node-metastasis, TNM）分期是判断NSCLC的进展程度及确定诊疗策略的重要指标^[2, 3]^。现有的TNM分期系统将N分期分为N0、N1、N2及N3，其中N2期NSCLC的异质性较大，不同淋巴结转移状态患者的预后差异很大^[4]^。国际抗癌联盟（Union for International Cancer Control, UICC）和美国癌症联合委员会（American Joint Committee on Cancer, AJCC）对NSCLC的TNM分期进行了多次修订，但N分期却鲜有变化。国际肺癌研究学会（International Association for the Study of Lung Cancer, IASLC）基于其肺癌数据库的病例信息，对不同N2亚组人群的预后差异进行分析，提出了N2亚分期^[5]^。尽管该亚分期已在许多研究中进行了验证，但尚未广泛应用于临床。本文拟对该亚分期的相关研究进行整理归纳，以明确其临床价值。

## 1 NSCLC的N2亚分期的定义

为了区分相同N分期中不同风险的NSCLC患者的预后，2007年IASLC将N分期进行了更细致的描述：根据淋巴结转移的站数，将N1期划分为单站N1淋巴结转移（N1a）和多站N1淋巴结转移（N1b）；将N2期划分为单站N2淋巴结转移（N2a）和多站N2淋巴结转移（N2b）。但该分类无法区分N2a与N1b人群的预后^[6]^。此后，IASLC在对NSCLC的第八版分期的建议中将N2a人群进一步分为N1淋巴结阴性的单站N2淋巴结转移（N2a1，即跳跃性单站N2淋巴结转移）和N1淋巴结阳性的单站N2淋巴结转移（N2a2）。该分类能更精细地区分N2期NSCLC患者的预后。

## 2 NCLC的N2亚分期人群的预后及复发模式

### 2.1 N1淋巴结阴性的单站N2淋巴结转移（N2a1）

#### 2.1.1 总生存期（overall survival, OS）

既往研究^[7-9]^显示，跳跃性N2淋巴结转移的预后优于非跳跃性，这提示在N2期NSCLC中，N1淋巴结转移仍对预后有一定的影响。此外，单站N2淋巴结转移的预后优于多站^[10-12]^。故兼具单站及跳跃性转移两个特点的N2a1期患者的预后较好。

IASLC对全球多中心的26, 236例患者的OS进行分析，结果^[5]^显示N2a1人群的OS显著优于N2a2人群（5年OS率：54% *vs* 43%，HR=0.74，*P* < 0.001），甚至在数值上优于N1b人群（5年OS率：54% *vs* 50%，HR=0.89，*P*=0.28）。表 1显示了各研究报道的不同亚分期人群的OS，其中病理分期为N2a1人群的5年OS率为36.3%-73.7%，而N2a2人群为21.5%-62.1%。大多数研究中N2a1人群的OS优于N2a2人群，其中8项研究显示这一差异具有统计学意义，其余研究可能因样本量的限制而无显著差异^[5, 13-29]^。表 1中病理分期为N1b人群的5年OS率为34.1%-64.3%，与N2a1人群相似；所有研究均显示N2a1人群的OS与N1b人群无显著差异，但大部分研究中N2a1人群的OS优于N1b人群。这些结果显示，仅根据淋巴结转移的解剖位置来确定N分期是不准确的，N2a1人群的OS显著优于N2a2人群，更接近甚至可能优于N1b人群。

**表 1:** NSCLC不同N2亚分期人群的OS情况 The OS of patients with different N2 sub-stages of NSCLC

Study (Author, Year)	Nation	Number of patients	Number of N2 patients	Types of staging	5-year OS rate (%)					The comparations of different sub-stages for OS		
					N1b	N2a1	N2a2	N2b		N2a1 *vs* N1b	N2a2 *vs* N2a1	N2b *vs* N2a2
Legras, 2014^[13]^	France	3, 910	817	Pathologic	-	36.3	27.8	-		-	-	-
Asamura, 2015^[5]^	Global	26, 236	1, 800	Pathologic	50	54	43	38		HR=0.89, *P*=0.28	HR=1.35, *P* < 0.001	HR=1.21, *P*=0.01
Bertoglio, 2018^[14]^	Italy	93	93	Pathologic	-	45.5	31.5	23.5		-	-	HR > 1, *P*=0.19
Chen, 2019^[15]^	China	570	152	Pathologic	39.3	38.7	41.7	15.3		*P*=0.97	*P*=0.99	*P* < 0.05
Park, 2019^[16]^	South Korea	1, 228	496	Pathologic	57	64.7	48.4	42.8		HR=0.82, *P*=0.27	HR=1.61, *P*=0.004	HR=1.58, *P*=0.004
Park, 2019^[17]^	South Korea	339	339	Pathologic	-	-	-	-		-	HR=1.11, *P*=0.37	HR=1.56, *P*=0.01
Yazgan, 2019^[18]^	Turkey	358	130	Pathologic	-	51.2	21.5	-		-	HR > 1, *P*=0.001	-
Yun, 2019^[19]^	South Korea	3, 971	606	Pathologic	51.7	61.8	47.9	34.6		HR=0.72, *P*=0.11	HR=1.60, *P*=0.003	HR=1.29, *P*=0.03
Chiappetta, 2020^[20]^	Italy	1, 036	477	Pathologic	49.6	44.8	32.8	-		HR=1.07, *P*=0.72	HR=1.39, *P*=0.04	HR > 1, *P* > 0.05
Kojima, 2020^[21]^	Japan	1, 581	194	Pathologic	49.9	73.7	62.1	46.9		HR=0.72, *P*=0.30	HR=1.50, *P*=0.14	HR=1.33, *P*=0.22
Yang, 2020^[22]^	China	773	773	Pathologic	-	48.9	40.4	-		-	HR=1.43, *P*=0.011	-
Aksoy, 2021^[23]^	Turkey	961	498	Pathologic	46.8	40.4	35.8	28.4		HR=1.10, *P*=0.53	HR=1.23, *P*=0.14	HR=1.20, *P*=0.13
Dziedzic, 2021^[24]*^	Poland	8, 016	910	Pathologic	34.1	37.3	32.4	-		HR=0.88, *P*=0.23	HR=1.13, *P*=0.02	-
Li, 2021^[25]^	China	642	337	Pathologic	64.3	62.2	56.5	37.3		HR=0.92, *P*=0.77	HR=1.31, *P*=0.26	HR=1.46, *P*=0.03
Tsitsias, 2021^[26]**^	U.K.	448	191	Pathologic	39.2 mon	33.3 mon	28.9 mon	24.6 mon		-	-	-
Abe, 2020^[27]^	Japan	34	34	Clinical	-	81.3	37.5	-		-	HR > 1, *P*=0.03	-
Song, 2021^[28]^	South Korea	1, 271	162	Clinical	36.0	29.2	34.4	18.0		HR=1.88, *P*=0.04	HR=0.98, *P*=0.93	HR=1.55, *P*=0.049
Stamatis, 2022^[29]^	Germany	145	145	y-pathologic	-	47.3	30.2	0		-	HR=1.89, *P*=0.009	HR > 1, *P* < 0.001
*This study reported the OS of patients with 6 or more nodes examined; **This study only reported the median OS but not the 5-year OS rate. OS: overall survival; NSCLC: non-small cell lung cancer.												

#### 2.1.2 无病生存期（disease-free survival, DFS）

IASLC未报道N2a1人群的DFS，后续研究对此进行了补充。表 2显示了各研究报道的不同亚分期人群的DFS，N2a1人群的5年DFS率为30.6%-54.0%，而N2a2人群为18.0%-38.2%。研究^[14, 16, 18, 19, 21-23, 27, 29]^显示，N2a1人群的DFS优于N2a2人群，其中6项研究具有统计学差异，其余研究可能因样本量的限制而无显著差异。表 2中N1b人群的5年DFS率为34.3%-43.6%，与N2a1人群相似；所有研究均显示N2a1人群的DFS优于N1b人群，其中Kojima等^[21]^的研究显示出统计学差异（HR=0.59, *P*=0.05）。这些结果显示，N2a1人群的复发风险低于N2a2人群，甚至可能低于N1b人群。

**表 2:** NSCLC不同N2亚分期人群的DFS情况 The DFS of patients with different N2 sub-stages of NSCLC

Study (Author, Year)	Nation	Number of patients	Number of N2 patients	Types of staging	5-year DFS rate (%)					The comparations of different sub-stages for DFS		
					N1b	N2a1	N2a2	N2b		N2a1 vs N1b	N2a2 vs N2a1	N2b *vs* N2a2
Bertoglio, 2018^[14]^	Italy	93	93	Pathologic	-	40	18	11		-	*P*=0.021	*P*=0.082
Park, 2019^[16]^	South Korea	1, 228	496	Pathologic	40.4	43.9	23.1	13.8		HR=0.88, *P*=0.45	HR=1.54, *P*=0.003	HR=1.45, *P*=0.008
Yazgan, 2019^[18]^	Turkey	358	130	Pathologic	-	44.7	21.4	-		-	HR > 1, *P*=0.001	-
Yun, 2019^[19]^	South Korea	3, 971	606	Pathologic	34.3	50.8	38.2	27.2		HR=0.75, *P*=0.15	HR=1.37, *P*=0.05	HR=1.37, *P*=0.01
Kojima, 2020^[21]^	Japan	1, 581	194	Pathologic	43.6	54.0	29.8	24.6		HR=0.59, *P*=0.05	HR=1.88, *P* < 0.01	HR=1.19, *P*=0.37
Yang, 2020^[22]^	China	773	773	Pathologic	-	38.4	27.6	-		-	HR=1.49, *P*=0.007	-
Aksoy, 2021^[23]^	Turkey	961	498	Pathologic	40.5	38.9	30.1	20.1		HR=1.06, *P*=0.70	HR=1.27, *P*=0.08	HR=1.32, *P*=0.02
Abe, 2020^[27]^	Japan	34	34	Clinical	-	53.6	29.6	-		-	HR > 1, *P*=0.13	-
Stamatis, 2022^[29]^	Germany	145	145	y-pathologic	-	30.6	23.4	0		-	HR=1.46, *P*=0.20	HR > 1, *P* < 0.001
DFS: disease-free survival.												

#### 2.1.3 复发模式

有关N2亚分期复发模式的研究有限，但仍有参考价值。Jin等^[30]^的研究纳入了440例N2期NSCLC患者，其中N2a1期未接受术后放疗的患者57例，其首发远处转移率及首发局部区域复发率分别为28.1%和12.3%，均低于N2a2期的42.0%和19.0%。Ohta等^[31]^的研究分析了N2a的NSCLC的复发模式，共纳入94例患者，其中N2a1患者的远处转移率及局部区域复发率分别为32%与12%，低于N2a2患者的52.0%和23.0%，该结果与Jin等^[30]^的研究有较好的一致性。此外，Wei等^[32]^的研究纳入了183例N2期NSCLC患者，将其分为N2a1、单站N1淋巴结阳性的单站N2淋巴结转移、多站N1淋巴结阳性的单站N2淋巴结转移以及N2b四组，结果显示N2a1患者的中位无局部区域复发生存期（locoregional-free survival, LRFS）显著优于其他三组患者，四组的中位LRFS分别为35个月、31个月、24个月及18个月（*P* < 0.001）。

此外，术后放疗对N2期NSCLC的复发模式有一定的影响^[33, 34]^，但这一效应在N2a1人群中不明显。前述Jin等^[30]^的研究中，接受术后放疗的41例N2a1患者的首发远处转移率及首发局部区域复发率分别为39.0%和9.8%；虽然数值比未放疗患者的首发远处转移率偏高、局部区域复发率偏低，但差异均无统计学意义。而Wei等^[32]^的研究也发现术后放疗不能显著改善N2a1患者的LRFS，术后放疗组与未放疗组的中位LRFS分别为39个月及33个月（*P*=0.97）。

总之，N2a1人群的复发模式以远处转移为主，其远处转移及局部区域复发风险均低于N2a2人群，术后放疗不能降低N2a1人群的局部区域复发。

### 2.2 N1淋巴结阳性的单站N2淋巴结转移（N2a2）

#### 2.2.1 OS

IASLC的全球多中心数据分析^[5]^结果显示，N2a2人群的预后显著优于N2b人群（5年OS率：54% *vs* 43%，HR=0.83，*P*=0.01）。表 1中病理分期为N2a2人群的5年OS率为21.5%-62.1%，而N2b人群的5年OS率为15.3%-46.9%。所有基于病理分期的研究都显示，N2a2人群的OS优于N2b人群，在5项研究中这一差异具有统计学意义，其余研究可能因样本量限制而无显著差异。而N2a2人群的OS劣于N2a1人群^[5, 16, 18-20, 22, 24, 27, 29]^。这些结果显示，尽管N2a2人群在单站N2淋巴结转移中预后较差，其OS依然优于N2b人群。

#### 2.2.2 DFS

IASL未报道N2a2人群的DFS，后续研究对此进行了补充。表 2中N2a2人群的5年DFS率为18.0%-38.2%，而N2b人群的5年DFS率为0%-27.2%。所有研究均显示，N2a2人群的DFS优于N2b人群，其中4项研究具有统计学差异，其余研究可能因样本量限制而无显著差异。而N2a2人群的DFS劣于N2a1人群^[14, 16, 18, 19, 21, 22]^。这说明N2a2人群的复发风险高于N2a1人群，低于N2b人群。

#### 2.2.3 复发模式

Jin等^[30]^的研究中纳入了100例未接受术后放疗的N2a2患者，其首发远处转移率及首发局部区域复发率分别为42.0%和19.0%；尽管N2a2患者的首发远处转移率低于N2b患者（42.0% *vs* 56.7%），但两类患者的首发局部区域复发率相近（19.0% *vs* 18.3%）。而N2a2患者的局部区域复发及远处转移风险均高于N2a1患者。

此外，术后放疗对N2a2 NSCLC局部区域复发的影响十分显著。Jin等^[30]^的研究中接受术后放疗的99例N2a2患者的首发远处转移率及首发局部区域复发率分别为41.4%和9.1%；术后放疗可以显著减少N2a2患者的首发局部区域复发（9.1% *vs* 19.0%, *P*=0.04），而不影响远处转移（41.4% *vs* 42.0%, *P*=0.99）；接受术后放疗的N2a2患者的首发局部区域复发率与N2a1患者相似（9.1% *vs* 9.8%）^[30]^。

总之，N2a2人群的复发模式以远处转移为主；尽管其远处转移风险较N2b人群低，但两者的局部区域复发相似；术后放疗可以显著改善N2a2人群的局部区域复发。

### 2.3 多站N2淋巴结转移（N2b）

#### 2.3.1 OS

N2b人群的OS劣于N2a2人群^[5, 15-17, 19, 25, 28, 29]^，但或许其内部存在低风险人群。在IASLC的亚分期基础上，许多研究试图根据有无跳跃性转移将N2b人群进一步分为N1淋巴结阴性的多站N2淋巴结转移（N2b1）与N1淋巴结阳性的多站N2淋巴结转移（N2b2）。表 3汇总了病理分期为N2b1及N2b2的NSCLC的OS情况^[13, 15, 22, 24]^。N2b1人群的5年OS率为26.3%-33.3%，N2b2人群为11.4%-24.4%。但仅Chen等^[15]^的研究中N2b1人群的OS显著优于N2b2（5年OS率：33.3% *vs* 11.4%，*P*=0.043）；Yang等^[22]^及Dziedzic等^[24]^的研究中N2b1与N2b2人群之间的OS无明显差异，这两项研究中N2b1与N2a2人群之间的OS也无明显差异。因此，N2b1与N2b2或N2a2人群的OS差异均不明显，N2b尚不足以划分亚分期，这可能与多站N2淋巴结转移的预后较差，掩盖了N1淋巴结转移对OS的影响有关。

**表 3:** 病理N2b1期及N2b2期NSCLC的OS情况 The OS of patients with pathological N2b1 and N2b2 NSCLC

Study (Author, Year)	Nation	Number of patients	Number of N2 patients	5-year OS rate (%)				The comparations of different sub-stages for OS	
				N2a2	N2b1	N2b2		N2b1 *vs* N2a2	N2b2 *vs* N2b1
Legras, 2014^[13]^	France	3, 910	817	27.8	26.3	14.3		-	-
Chen, 2019^[15]^	China	570	152	41.7	33.3	11.4		-	HR > 1, *P*=0.043
Yang, 2020^[22]^	China	773	773	40.4	26.7	24.4		HR=1.13, *P*=0.45	HR=1.25, *P*=0.18
Dziedzic, 2021^[24]*^	Poland	8, 016	910	32.4	29.4	23.0		HR=1.09, *P*=0.53	HR=1.25, *P*=0.14
*This study reported the OS of patients with 6 or more nodes examined.									

#### 2.3.2 DFS

多站淋巴结转移的治疗失败风险高于单站^[35, 36]^，N2b人群的DFS较N2a2人群更差^[16, 19, 23, 29]^，一些研究试图根据DFS差异找到N2b中的低风险人群。Yang等^[22]^的研究纳入773例N2期NSCLC，以比较N2b1、N2a2及N2b2人群的DFS，结果N2b1与N2b2人群的5年DFS率没有明显差异（20.9% *vs* 12.0%, HR=1.27, *P*=0.13），与N2a2人群也没有明显差异（20.9% *vs* 27.6%, HR=0.81, *P*=0.21）。因此，将N2b人群继续划分亚分期目前没有临床意义。

#### 2.3.3 复发模式

Jin等^[30]^的研究纳入了60例未接受术后放疗的N2b期患者，其首发远处转移率及首发局部区域复发率分别为56.7%与18.3%。而一些不涉及亚分期的研究也报道了多站N2转移患者的局部区域复发情况，Han等^[37]^的研究中113例多站N2患者的3年局部区域控制率为41.0%，低于单站N2患者的61.5%（*P*=0.037）。Ichinose等^[38]^的研究中162例多站N2患者的5年无局部区域复发率（freedom from local recurrence, FFLR）为47.9%，低于单站N2患者的75.2%（*P* < 0.001）。

此外，术后放疗对N2b患者的局部区域复发的影响十分显著，Jin等^[30]^的研究中接受术后放疗的83例N2b患者的首发远处转移率及首发局部区域复发率分别为65.0%和7.2%。术后放疗可以显著减少N2b患者的首发局部区域复发率（7.2% *vs* 18.3%, *P*=0.04），而不影响远处转移（65.0% *vs* 56.7%, *P*=0.31）。而Wei等^[32]^的研究也发现术后放疗可以显著改善N2b患者的LRFS，术后放疗组与未放疗组的中位LRFS分别为27个月和11个月（*P* < 0.001）。

总之，N2b人群的复发模式以远处转移为主，超过一半的患者在5年内出现远处转移，术后放疗对N2b人群的远处转移没有影响，但可以显著改善局部区域复发。

## 3 总结

IASLC建议的N2亚分期可以更好地区分NSCLC的OS、DFS及复发模式。N2a1人群的OS及DFS更接近N1b；N2a2人群的OS及DFS介于N2a1及N2b之间；N2b人群的OS及DFS较差，目前的证据尚不足以将N2b进一步划分。尽管危险度不同，但N2a1、N2a2及N2b三个亚分期的主要复发模式均为远处转移，且远处转移的风险依次升高。N2a1人群的局部区域复发风险较低，术后放疗不能改善其局部区域复发；而N2a2和N2b人群的局部区域复发风险相似，术后放疗可以显著改善这两个人群的局部区域复发，使之降低至与N2a1人群相近的水平。

根据目前数据综合分析，本文对NSCLC的N分期提出以下改良意见：①将N2a1人群划入N1期；②将N2a2期、N2b期作为两个独立的N2亚分期；③N2b期暂不进一步划分。这有助于更精细区分N2期NSCLC患者的预后，制定精准的治疗策略。
